# Iatrogenic carpal tunnel syndrome induced by wrist extension for placement of an indwelling radial artery catheter: a case report

**DOI:** 10.1186/s40981-017-0120-7

**Published:** 2017-09-18

**Authors:** Kunitaro Watanabe, Shingo Mitsuda, Akira Motoyasu, Joho Tokumine, Kumi Moriyama, Tomoko Yorozu

**Affiliations:** Department of Anesthesiology, Kyorin University School of Medicine, 6-20-2 Shinkawa, Mitaka-shi, Tokyo 181-8611 Japan

**Keywords:** Radial artery catheter, Carpal tunnel syndrome, Complication

## Abstract

A 38-year-old man with pancreatic cancer was scheduled to undergo pancreaticoduodenectomy. He had an unremarkable past medical history. After inducing general anesthesia, a left radial arterial catheter was successfully placed at first attempt. A wrist splint was used to obtain good arterial pulse waveforms.

After the operation, he was transferred to the intensive care unit. The radial artery catheter was removed on the fourth postoperative day. He experienced numbness and a tingling sensation in the left thumb, the second and third fingers, and the lateral half of the fourth finger. He was diagnosed with carpal tunnel syndrome. Diagnostic imaging revealed a swollen median nerve, but no hematoma or injury.

Some studies have suggested that excessive extension of the wrist may cause neuropathy. We recommend that patients’ wrists not be over-extended, even if good arterial waveforms cannot be obtained.

**Electronic supplementary material:**

The online version of this article (10.1186/s40981-017-0120-7) contains supplementary material, which is available to authorized users.

## Background

The radial artery is the most common site for arterial cannulation, and radial artery catheterization is a relatively safe procedure for continuous intraoperative measurement of arterial pressure. Serious complications of radial artery catheterization occur in less than 0.2% [1]. The complications reported include occlusion of the radial artery, hematoma, and nerve injury [2]. However, complications related to radial artery catheterization are not limited to those owing to direct injury during the procedure [3, 4]. We report a case of iatrogenic carpal tunnel syndrome induced by wrist extension for indwelling radial artery catheter placement.

## Case presentation

A 38-year-old man was diagnosed with pancreatic cancer and was scheduled to undergo pancreaticoduodenectomy. He had an unremarkable medical history. A combination of general anesthesia and epidural anesthesia was considered. Epidural tubing was performed at T8/9, and general anesthesia was induced with 100 μg fentanyl, 100 mg propofol, and 50 mg rocuronium. They were administered intravenously through a peripheral venous catheter inserted in his left cephalic vein near the wrist.

Radial artery catheterization was planned for measuring continuous arterial pressure during surgery. A left radial arterial line was successfully placed on the first try. A wrist split was used to obtain a good arterial pulse waveform.

The surgery was completed on schedule. The total operation time was 7 h 55 min. After the surgery, he was transferred to an intensive care unit (ICU) where he stayed for 4 days and had a wrist-extension split placed.

The radial artery catheter was removed when he was transferred to a general ward. He experienced numbness and a tingling sensation in his left palm during his ICU stay that he considered transient and insignificant and did not report it to the ICU staff. This continued after ICU discharge. On the 10th postoperative day, he complained about his numbness to the physician in charge. The physician consulted a pain specialist, and the medical examination revealed that numbness and paresthesia were present in the left thumb, the second and third fingers, and the lateral half of the fourth finger. His left hand had a weak grip. The patient doubted that these symptoms resulted from an incident during placement of the indwelling radial artery catheter because he recalled irritable lightning and burning pain in his left wrist during the induction of general anesthesia.

**Fig. 1:**
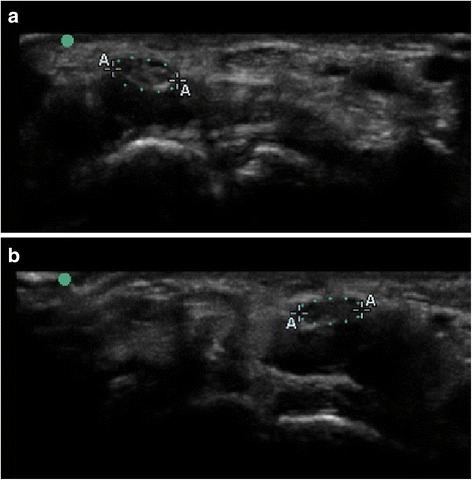
Ultrasound transverse view of the patient’s wrist. **a** Left wrist (the palm of which suffered from numbness and tingling). **b** Right wrist. Dashed circles indicate the median nerves. The figure shows enlargement of the left median nerve compared to the right median nerve

Ultrasound examination revealed no hematomas and a normal appearance of the radial artery and radial nerve, but an enlargement of the left median nerve compared with the right median nerve at the pisiform bone level was observed (cross section areas of the left and right median nerves were 12 and 9 mm^2^, respectively) (Fig. 1). The physical examination showed a positive Tinel’s sign, positive Phalen’s sign (20 s), and positive Flick sign. Considering the present findings, carpal tunnel syndrome was strongly suspected.

**Fig. 2:**
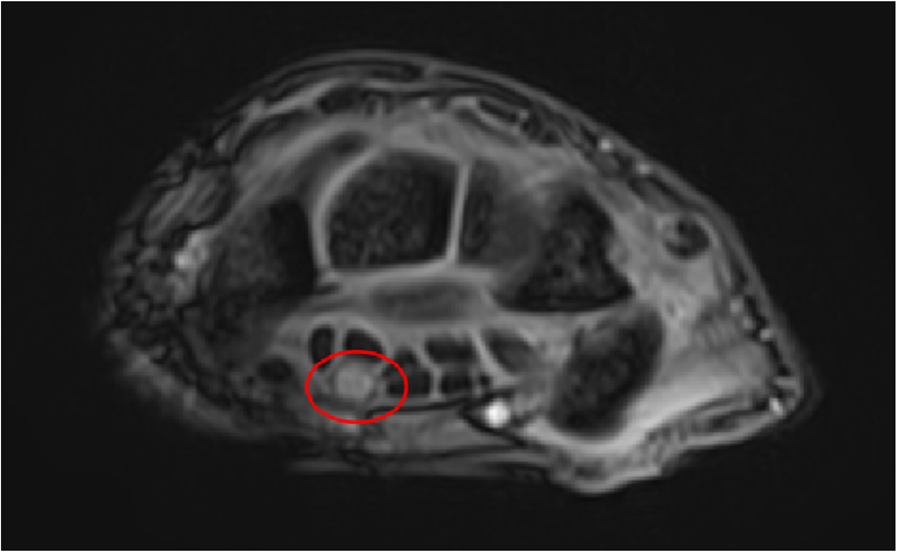
Magnetic resonance imaging of the left wrist. The red circle indicates the left median nerve. Magnetic resonance imaging shows swelling of the left median nerve and no hematoma

Contrast-enhanced computed tomography (CT) and magnetic resonance imaging (MRI) were performed. There were no hematomas and no abnormality or deformation of the radial artery and carpal bones on contrast-enhanced CT. There was an enlargement of the median nerve at the pisiform bone level on fat suppression T2-weighted MRI (Fig. 2). There was no thickening of the flexor tendon, but there was fluid stagnation in the deep part of the bursa and intense signals around the flexor tendon; these are typical findings in carpal tunnel syndrome even though the patient has no past medical history of carpal tunnel syndrome. During interviews, some ICU nurses recalled that his wrist-extension split had been fixed in a relatively hyperextended position for 4 days to obtain a good arterial pulse waveform.

Neurological diagnosis revealed iatrogenic carpal tunnel syndrome due to hyperextension of the wrist. The patient’s recollection of burning pain in his left wrist during induction of general anesthesia was considered a side effect of propofol. We started to administer 75 mg pregabalin daily and injected 4 mg of dexamethasone steroid into the carpal canal. He was discharged on the 12th postoperative day. The numbness in his palm interfered with his ability to type and impacted his quality of life. However, it gradually subsided. He was medicated and followed up at the outpatient pain clinic for 6 months before he made a complete recovery.

## Discussion

There are only two published reports of carpal tunnel syndrome caused by radial artery cannulation. They consisted of an iatrogenic carpal tunnel syndrome and carpal tunnel syndrome with sequelae of complex regional pain syndrome. In both reports, neuropathies were due to hematomas, whereas our reported iatrogenic carpal tunnel syndrome was not related to hematomas [3, 4]. Our patient had none of the risk factors for carpal tunnel syndrome such as diabetes, rheumatism, or amyloidosis. To our knowledge, this is the first report of carpal tunnel syndrome induced by radial artery cannulation without hematomas.

Chowet et al. reported that hyperextension of the wrist for 60 min could induce sensory loss in the palm [5]. The mechanism responsible is compression of the median nerve due to the severely extended position of the wrist may [6]. In our case, the long-term extension of the wrist may have induced the carpal tunnel syndrome.

Ultrasonography may be useful as an alternative diagnostic examination, especially as it can be employed more quickly than MRI [7, 8]. However, the clinical efficacy and diagnostic reliability of ultrasonography in carpal tunnel syndrome has not yet been established and further studies are needed.

Recently, there have been reports on the efficacy of ultrasound guidance during radial artery catheterization. Ultrasound-guided radial artery catheterization increases the chances of success on the first attempt and reduces complications, such as hematoma, compared to the traditional palpation landmark technique [9, 10]. We emphasize that ultrasound-guided radial artery catheterization can be performed more centrally on the forearm than the usual palpation technique (Additional file 1). Placing the indwelling radial artery catheter more than 1 in. away from the wrist has the benefit of not necessitating a wrist extension split. Furthermore, this may contribute towards improving the patient’s quality of life in the ICU.

The question of why the ICU staff did not notice the symptoms in the patient’s hand for 4 days remains. During the medical examination by the pain specialist, the patient stated that he noticed numbness and a tingling sensation in his left palm during his ICU stay. However, he did not report his symptoms to the ICU staff. If the ICU staff had asked the patient about his symptoms directly, they would have been noticed easily. We speculated that the reason was closely related to the specific character of carpal tunnel syndrome. The same specific symptoms can be found in another disease: anterior interosseous nerve palsy, also known as “honeymoon palsy” [11], where compression of the nerve causes the same symptoms. In this disease, the affected patient does not notice his numbness until the morning after the wedding night. To discover the symptoms of carpal tunnel syndrome, the ICU staff should ask the patient about them directly. For this to be effective, the ICU staff should have sufficient knowledge of this syndrome, especially the fact that it may be caused by an over-extended wrist split.

## Conclusions

Early detection of carpal tunnel syndrome caused by over-extension of the wrist may help reduce its severity and alleviate the patient’s symptoms. However, to avoid this complication, ultrasound guidance and catheter insertion 1 in. away from the wrist during radial artery catheterization is recommended.

## Additional file

Additional file 1: Ultrasound-guided radial artery catheterization. (MP4 16,178 kb)
